# Open Targets Genetics: systematic identification of trait-associated genes using large-scale genetics and functional genomics

**DOI:** 10.1093/nar/gkaa840

**Published:** 2020-10-12

**Authors:** Maya Ghoussaini, Edward Mountjoy, Miguel Carmona, Gareth Peat, Ellen M Schmidt, Andrew Hercules, Luca Fumis, Alfredo Miranda, Denise Carvalho-Silva, Annalisa Buniello, Tony Burdett, James Hayhurst, Jarrod Baker, Javier Ferrer, Asier Gonzalez-Uriarte, Simon Jupp, Mohd Anisul Karim, Gautier Koscielny, Sandra Machlitt-Northen, Cinzia Malangone, Zoe May Pendlington, Paola Roncaglia, Daniel Suveges, Daniel Wright, Olga Vrousgou, Eliseo Papa, Helen Parkinson, Jacqueline A L MacArthur, John A Todd, Jeffrey C Barrett, Jeremy Schwartzentruber, David G Hulcoop, David Ochoa, Ellen M McDonagh, Ian Dunham

**Affiliations:** Wellcome Sanger Institute, Wellcome Genome Campus, Hinxton, Cambridgeshire CB10 1SA, UK; Open Targets, Wellcome Genome Campus, Hinxton, Cambridgeshire CB10 1SD, UK; Wellcome Sanger Institute, Wellcome Genome Campus, Hinxton, Cambridgeshire CB10 1SA, UK; Open Targets, Wellcome Genome Campus, Hinxton, Cambridgeshire CB10 1SD, UK; Open Targets, Wellcome Genome Campus, Hinxton, Cambridgeshire CB10 1SD, UK; European Molecular Biology Laboratory, European Bioinformatics Institute (EMBL-EBI), Wellcome Genome Campus, Hinxton, Cambridgeshire CB10 1SD, UK; Open Targets, Wellcome Genome Campus, Hinxton, Cambridgeshire CB10 1SD, UK; European Molecular Biology Laboratory, European Bioinformatics Institute (EMBL-EBI), Wellcome Genome Campus, Hinxton, Cambridgeshire CB10 1SD, UK; Wellcome Sanger Institute, Wellcome Genome Campus, Hinxton, Cambridgeshire CB10 1SA, UK; Open Targets, Wellcome Genome Campus, Hinxton, Cambridgeshire CB10 1SD, UK; Open Targets, Wellcome Genome Campus, Hinxton, Cambridgeshire CB10 1SD, UK; European Molecular Biology Laboratory, European Bioinformatics Institute (EMBL-EBI), Wellcome Genome Campus, Hinxton, Cambridgeshire CB10 1SD, UK; Open Targets, Wellcome Genome Campus, Hinxton, Cambridgeshire CB10 1SD, UK; European Molecular Biology Laboratory, European Bioinformatics Institute (EMBL-EBI), Wellcome Genome Campus, Hinxton, Cambridgeshire CB10 1SD, UK; Open Targets, Wellcome Genome Campus, Hinxton, Cambridgeshire CB10 1SD, UK; European Molecular Biology Laboratory, European Bioinformatics Institute (EMBL-EBI), Wellcome Genome Campus, Hinxton, Cambridgeshire CB10 1SD, UK; Open Targets, Wellcome Genome Campus, Hinxton, Cambridgeshire CB10 1SD, UK; European Molecular Biology Laboratory, European Bioinformatics Institute (EMBL-EBI), Wellcome Genome Campus, Hinxton, Cambridgeshire CB10 1SD, UK; Open Targets, Wellcome Genome Campus, Hinxton, Cambridgeshire CB10 1SD, UK; European Molecular Biology Laboratory, European Bioinformatics Institute (EMBL-EBI), Wellcome Genome Campus, Hinxton, Cambridgeshire CB10 1SD, UK; Open Targets, Wellcome Genome Campus, Hinxton, Cambridgeshire CB10 1SD, UK; European Molecular Biology Laboratory, European Bioinformatics Institute (EMBL-EBI), Wellcome Genome Campus, Hinxton, Cambridgeshire CB10 1SD, UK; Open Targets, Wellcome Genome Campus, Hinxton, Cambridgeshire CB10 1SD, UK; European Molecular Biology Laboratory, European Bioinformatics Institute (EMBL-EBI), Wellcome Genome Campus, Hinxton, Cambridgeshire CB10 1SD, UK; Open Targets, Wellcome Genome Campus, Hinxton, Cambridgeshire CB10 1SD, UK; European Molecular Biology Laboratory, European Bioinformatics Institute (EMBL-EBI), Wellcome Genome Campus, Hinxton, Cambridgeshire CB10 1SD, UK; Open Targets, Wellcome Genome Campus, Hinxton, Cambridgeshire CB10 1SD, UK; European Molecular Biology Laboratory, European Bioinformatics Institute (EMBL-EBI), Wellcome Genome Campus, Hinxton, Cambridgeshire CB10 1SD, UK; Open Targets, Wellcome Genome Campus, Hinxton, Cambridgeshire CB10 1SD, UK; European Molecular Biology Laboratory, European Bioinformatics Institute (EMBL-EBI), Wellcome Genome Campus, Hinxton, Cambridgeshire CB10 1SD, UK; Open Targets, Wellcome Genome Campus, Hinxton, Cambridgeshire CB10 1SD, UK; European Molecular Biology Laboratory, European Bioinformatics Institute (EMBL-EBI), Wellcome Genome Campus, Hinxton, Cambridgeshire CB10 1SD, UK; Wellcome Sanger Institute, Wellcome Genome Campus, Hinxton, Cambridgeshire CB10 1SA, UK; Open Targets, Wellcome Genome Campus, Hinxton, Cambridgeshire CB10 1SD, UK; Open Targets, Wellcome Genome Campus, Hinxton, Cambridgeshire CB10 1SD, UK; GlaxoSmithKline plc, GSK Medicines Research Centre, Gunnels Wood Road, Stevenage SG1 2NY, UK; Open Targets, Wellcome Genome Campus, Hinxton, Cambridgeshire CB10 1SD, UK; GlaxoSmithKline plc, GSK Medicines Research Centre, Gunnels Wood Road, Stevenage SG1 2NY, UK; Open Targets, Wellcome Genome Campus, Hinxton, Cambridgeshire CB10 1SD, UK; European Molecular Biology Laboratory, European Bioinformatics Institute (EMBL-EBI), Wellcome Genome Campus, Hinxton, Cambridgeshire CB10 1SD, UK; Open Targets, Wellcome Genome Campus, Hinxton, Cambridgeshire CB10 1SD, UK; European Molecular Biology Laboratory, European Bioinformatics Institute (EMBL-EBI), Wellcome Genome Campus, Hinxton, Cambridgeshire CB10 1SD, UK; Open Targets, Wellcome Genome Campus, Hinxton, Cambridgeshire CB10 1SD, UK; European Molecular Biology Laboratory, European Bioinformatics Institute (EMBL-EBI), Wellcome Genome Campus, Hinxton, Cambridgeshire CB10 1SD, UK; Open Targets, Wellcome Genome Campus, Hinxton, Cambridgeshire CB10 1SD, UK; European Molecular Biology Laboratory, European Bioinformatics Institute (EMBL-EBI), Wellcome Genome Campus, Hinxton, Cambridgeshire CB10 1SD, UK; Wellcome Sanger Institute, Wellcome Genome Campus, Hinxton, Cambridgeshire CB10 1SA, UK; Open Targets, Wellcome Genome Campus, Hinxton, Cambridgeshire CB10 1SD, UK; Open Targets, Wellcome Genome Campus, Hinxton, Cambridgeshire CB10 1SD, UK; European Molecular Biology Laboratory, European Bioinformatics Institute (EMBL-EBI), Wellcome Genome Campus, Hinxton, Cambridgeshire CB10 1SD, UK; Open Targets, Wellcome Genome Campus, Hinxton, Cambridgeshire CB10 1SD, UK; Systems Biology, Biogen, Cambridge, MA 02142, USA; Open Targets, Wellcome Genome Campus, Hinxton, Cambridgeshire CB10 1SD, UK; European Molecular Biology Laboratory, European Bioinformatics Institute (EMBL-EBI), Wellcome Genome Campus, Hinxton, Cambridgeshire CB10 1SD, UK; European Molecular Biology Laboratory, European Bioinformatics Institute (EMBL-EBI), Wellcome Genome Campus, Hinxton, Cambridgeshire CB10 1SD, UK; Wellcome Centre for Human Genetics, Nuffield Department of Medicine, NIHR Oxford Biomedical Research Centre, University of Oxford, Roosevelt Drive, Oxford OX3 7BN, UK; Wellcome Sanger Institute, Wellcome Genome Campus, Hinxton, Cambridgeshire CB10 1SA, UK; Open Targets, Wellcome Genome Campus, Hinxton, Cambridgeshire CB10 1SD, UK; Wellcome Sanger Institute, Wellcome Genome Campus, Hinxton, Cambridgeshire CB10 1SA, UK; Open Targets, Wellcome Genome Campus, Hinxton, Cambridgeshire CB10 1SD, UK; Open Targets, Wellcome Genome Campus, Hinxton, Cambridgeshire CB10 1SD, UK; GlaxoSmithKline plc, GSK Medicines Research Centre, Gunnels Wood Road, Stevenage SG1 2NY, UK; Open Targets, Wellcome Genome Campus, Hinxton, Cambridgeshire CB10 1SD, UK; European Molecular Biology Laboratory, European Bioinformatics Institute (EMBL-EBI), Wellcome Genome Campus, Hinxton, Cambridgeshire CB10 1SD, UK; Wellcome Sanger Institute, Wellcome Genome Campus, Hinxton, Cambridgeshire CB10 1SA, UK; European Molecular Biology Laboratory, European Bioinformatics Institute (EMBL-EBI), Wellcome Genome Campus, Hinxton, Cambridgeshire CB10 1SD, UK; Wellcome Sanger Institute, Wellcome Genome Campus, Hinxton, Cambridgeshire CB10 1SA, UK; Open Targets, Wellcome Genome Campus, Hinxton, Cambridgeshire CB10 1SD, UK; European Molecular Biology Laboratory, European Bioinformatics Institute (EMBL-EBI), Wellcome Genome Campus, Hinxton, Cambridgeshire CB10 1SD, UK

## Abstract

Open Targets Genetics (https://genetics.opentargets.org) is an open-access integrative resource that aggregates human GWAS and functional genomics data including gene expression, protein abundance, chromatin interaction and conformation data from a wide range of cell types and tissues to make robust connections between GWAS-associated loci, variants and likely causal genes. This enables systematic identification and prioritisation of likely causal variants and genes across all published trait-associated loci. In this paper, we describe the public resources we aggregate, the technology and analyses we use, and the functionality that the portal offers. Open Targets Genetics can be searched by variant, gene or study/phenotype. It offers tools that enable users to prioritise causal variants and genes at disease-associated loci and access systematic cross-disease and disease-molecular trait colocalization analysis across 92 cell types and tissues including the eQTL Catalogue. Data visualizations such as Manhattan-like plots, regional plots, credible sets overlap between studies and PheWAS plots enable users to explore GWAS signals in depth. The integrated data is made available through the web portal, for bulk download and via a GraphQL API, and the software is open source. Applications of this integrated data include identification of novel targets for drug discovery and drug repurposing.

## INTRODUCTION

The identification of novel druggable targets for developing safe and effective medicines is a key priority for the pharmaceutical industry. However, drug development is inefficient, with over 90% of the drugs that enter clinical trials failing, often at late stages, with the primary reason being an inability to demonstrate efficacy (1). These failures inflict major resource and time losses where it is estimated that the total R&D cost per new drug is over $2.5 billion (2). They also largely reflect our poor understanding of disease biology and hence it is critical to incorporate new evidence in drug development decisions that could help impact drug success. Drugs with targets that have underlying genetic evidence for disease association have been shown to be twice as likely to succeed in clinical development (3,4). Therefore, systematic evaluation of genetic associations for a particular disease or trait can aid discovery of suitable targets (genes) for drug development. There is now a large and ever-growing number of human genome-wide association studies (GWAS) and population biobank studies generating evidence for the role of genetic variants in common or complex disease phenotypes.

The majority of GWAS-associated variants fall in the non-coding part of the genome suggesting that they affect complex traits and diseases through altering expression of neighbouring genes using regulatory mechanisms. Identifying the causal gene underlying each association signal is an intense process requiring the integration of data from GWAS with transcriptomics, proteomics and epigenomics datasets from a wide range of cell types and tissues. Experimental validation is then used to confirm the likely causal variant(s) and the gene it is regulating. International efforts and consortia have used integrative approaches to identify target genes for specific therapy areas but there are currently no resources that leverage publicly available GWAS and functional genomics data in a scalable and reproducible way to systematically assign causal variants and target genes at all disease-associated loci.

In the absence of a publicly available portal that helps address these challenges and allows a wide array of biological questions to be answered systematically, we developed Open Targets Genetics (https://genetics.opentargets.org), an open-access integrative resource that enables statistical genetics and functional genomics scientists to translate signals from Genome Wide Association Studies (GWAS) and Biobank data into target gene(s) across thousands of traits genome-wide using a workflow suited to their needs. This portal was constructed based on the latest technology to enable data to be easily added and browsed. It contains genetic and functional genomics data from a wide range of repositories and datasets, as well as additional data analyses generated internally using robust statistical methods. The data integrated into Open Targets Genetics can be searched by gene, variant or trait/disease. It offers several features that are fundamental for refining GWAS signals and identifying the likely causal variant(s) and target gene(s) at trait-associated loci. These include: (i) systematic statistical fine-mapping across thousands of trait-associated loci to resolve association signals, (ii) cross-trait/disease-disease colocalisation analysis and disease-molecular trait colocalisation analysis across 92 tissues and cell types, (iii) linking each variant to its likely target gene(s), using a single evidence score aggregating across a range of functional genomics datasets (disease agnostic), (iv) prediction of the likely causal gene(s) for all GWAS-associated loci using a machine learning model combining genetics and functional genomics information in a single ‘locus to gene’ (L2G) score, (v) Phenome Wide Association Study (PheWAS) analysis across a wide range of diseases and traits, (vi) enriched trait—gene evidence with orthogonal evidence including clinical trials that might support therapeutic hypothesis by linking with the Open Targets Platform (https://www.targetvalidation.org/) (5,6).

This stand-alone genetics resource within the Open Targets ecosystem complements the existing Open Targets Platform and provides underlying genetic evidence to aid drug target identification and prioritisation.

### Overview of the Open Targets Genetics Portal

**Figure 1.**
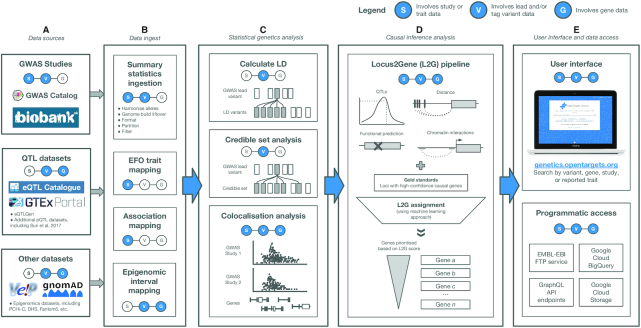
Data resources, ingestion process, data analyses and accessibility of the Open Target Genetics Portal. (**A**) The datasets ingested into Open Targets Genetics, (**B**) the data ingest and mapping processes, (**C**) statistical genetics analyses carried out, (**D**) the Locus to Gene pipeline (L2G), (**E**) accessibility options for users. Abbreviations: API; Application Programming Interface, EFO; experimental factor ontology, EMBL-EBI; European Molecular Biology Laboratory – European Bioinformatics Institute, LD; linkage disequilibrium, FTP; file transfer protocol.

Figure 1 outlines Open Targets Genetics Portal: the publicly-available data sources (A), the processes required to ingest and integrate the datasets together (B), the internal statistical genetics and causal inference data analyses carried out to provide additional information to users (C and D), and the available access options (E).

### Data resources

Several sources of GWAS data are integrated into the portal in order to establish the link between variants and traits/diseases (Figure 1A). Firstly, GWAS studies with summary statistics are retrieved from the NHGRI-EBI GWAS Catalog summary statistics database (*N* = 300 currently) (7). Due to a lack of suitable reference genotypes for conditional analysis required for non-European populations, full summary statistics are only used for studies that are predominantly of European ancestry. Although most studies to date have been in European populations, we aim to expand the scope of studies in Open Targets Genetics to new populations as soon as large enough reference panels become available. Secondly, two published GWAS analyses utilising UK Biobank data have been integrated: the SAIGE study of 2139 binary (case-control) phenotypes, and the Neale lab study of 1283 quantitative traits (http://www.nealelab.is/uk-biobank) (8). Ingestion of summary statistics includes harmonisation of alleles, genome build liftover, formatting, partitioning and filtering (Figure 1B). Lastly, studies from the NHGRI-EBI GWAS Catalog which do not have summary statistics are also included, adding a further 14 013 studies (7).

In addition to GWAS, functional genomics data provides evidence for the effect of variants on genes (Figure 1A). Molecular phenotype quantitative trait loci data (QTLs) includes protein QTL (pQTL) data of 2994 plasma proteins assessed in 3301 individuals of European descent (9). Gene expression QTLs (eQTLs) are integrated from the eQTL Catalogue, eQTLGen and GTEx (https://www.gtexportal.org/home/) (10,11). Other datasets that provide evidence for variant to gene (V2G) association include epigenetics chromatin conformation and interaction experiments with promoter capture hiC (PCHI-C) from 27 different cell types, enhancer-TSS pairwise cap analysis of gene expression (CAGE) correlation, and DNase I hypersensitive site (DHS)-gene promoter correlation (12–14). Each epigenetic data point is represented as a pair of interacting genomic intervals and an association statistic. Interval pairs are retained with one end encompassing an Ensembl gene Transcription Start Site (TSS) and the other end containing any variant in GnomAD 2.1 (Figure 1B) (15,16).

All traits have been manually mapped to the Experimental Factor Ontology (EFO) (17). This level of data standardisation provides additional value for users interested in integrating data across studies.

### Statistical genetics analysis

In addition to the large resource of genetics and functional genomics datasets, Open Targets Genetics also analyses the available evidence in order to link variants to disease, variants to genes and ultimately genes to disease. This, for example, enables prioritisation of causal genes for a specific disease based on robust genetic and functional genomics information, thus allowing better prioritisation of potential drug targets.

We use the following data model:

$$\begin{eqnarray*}{\rm Study }({\rm S})&-&{\rm Lead\, Variant }({\rm V}_{\rm L}) - {\rm Tag\,Variant}({\rm V}_{\rm T})\nonumber\\ &-& {\rm Gene}({\rm G})\end{eqnarray*}$$

Disease and trait association information (Study) are obtained from the GWAS which links disease status (or other trait measurements) to common genetic variation. A common association significance threshold of *P* < 5e–8 is set for all studies. While some studies provide the complete summary statistics, others only report the lead variant (V_L_) at each associated locus. However, it cannot be assumed that the V_L_ is causing the association; due to linkage disequilibrium (LD), the V_L_ might belong to a set of SNPs that have travelled together with the true causal SNP on a haplotype block. For this reason, fine-mapping/credible set analysis and LD expansion are implemented to include all tag variants (V_T_) and provide a more comprehensive set of potentially *causal* variants linked to the trait (Figure 1C).

Given that not all GWAS have summary statistics available, two fine-mapping methods are applied, one using full summary statistics, and another using LD information only. For studies with full summary statistics, we use GCTA-COJO to identify independent signals, and then perform per-signal conditional analysis adjusting for other independent signals in a ±2 Mb region from the lead variant (18). For every conditionally independent signal, fine-mapping using the Approximate Bayes Factor approach is performed (19). For GWAS without summary statistics, we use the PICS method with an LD reference from the most closely matched 1000 genomes project superpopulation (20). This enables us to estimate the probability that each variant is causal across 133,441 study-lead variant associated loci. Output from both methods provides a posterior probability (PP) for each variant being causal for a given association. In order to identify complex traits and diseases that share common molecular mechanisms, we also perform cross-trait colocalization analyses for 3,621 GWAS studies with summary statistics.

To assign likely causal genes for a given variant, we initially developed a disease-agnostic Variant to Gene (V2G) analysis pipeline which provides a single aggregated score for each variant-gene prediction. This analysis combines four different data types; molecular phenotype quantitative trait loci datasets (eQTLs and pQTLs), chromatin interaction and conformation datasets, *in silico* functional predictions (using the Variant Effect Predictor or VEP score), and distance from the canonical transcript start site (21). The data harmonisation and aggregation process as well as the weighting applied to each of the datasets are described here: https://genetics-docs.opentargets.org/our-approach/data-pipeline. More recently, we have developed a disease-specific gene prioritisation approach (Locus to Gene score, L2G) to prioritise genes at all trait-associated loci using a machine learning model. For this, we integrate fine-mapping credible set analysis across all 133,441 loci with functional genomics data (including pathogenicity prediction, colocalisation with molecular quantitative trait loci, genomic distance and chromatin interaction data) to generate L2G predictive features. We then train a supervised model using over 400 gold-standard positive GWAS loci for which we are confident of the gene implicated to predict causal genes at each locus (see https://github.com/opentargets/genetics-gold-standards). It is important to note that the existing gold-standard genes are likely to be biased towards those that are near the centre of the GWAS peak and which have clear (nonsynonymous) variant consequences, which will influence the features learned in the L2G model. We intend to continue expanding the repository of gold standard loci to enable building the most accurate model possible for gene prioritisation. More details on the machine learning method are described in available online documentation (https://genetics-docs.opentargets.org/our-approach/pipeline-overview).

To summarise, three statistical genetics data analyses are carried out as outlined in Figure 1C. Lead variant annotation, and lead variant to tag variant expansion methods, which include fine-mapping/credible set analysis and linkage-disequilibrium expansion are described in full here: https://genetics-docs.opentargets.org/our-approach/assigning-traits-to-loci. Disease-molecular trait colocalisation analysis for studies with full summary statistics is explained in more detail here: https://genetics-docs.opentargets.org/our-approach/colocalisation-analysis. Similar analyses have been conducted for GWAS studies without full summary statistics, using an approximate colocalisation heuristic based on variant probabilities from the PICS method. These analyses then feed into the causal inference L2G analysis pipeline to connect the associated loci to genes, utilising underlying evidence in order to ultimately rank the genes most likely to be underlying the associated trait/disease (Figure 1C).

### A technical look into the Genetics Portal

Retrieving, processing, analysing and presenting the large amount of biological data in the Genetics Portal introduces some challenges. The majority of the data currently in the Portal corresponds to public information that we downloaded from the respective resources and analysed in Google Cloud Platform (GCP). Other datasets such as the UK Biobank LD reference panel have more restrictive access conditions and so were analysed locally. Storing and processing the hundreds of TB of raw data has also required some technical solutions for large scale data manipulation. Other challenges relate to the algorithmic part of the analysis. For example, the full cross-trait and QTL colocalisation currently takes about 4 weeks, conducting 2,035,470 successful comparisons on a compute cluster using 60 CPU cores. Since computing pairwise similarities is an O(*N*^2^) problem, we are looking into new methods that might alleviate the current constraints. Considering the speed at which population genomics data is currently generated, maintaining a set of reference datasets (e.g. LD panels, variant indexes) also introduces the need for keeping infrastructure up-to-date.

All data is harmonised, analysed and merged using a combination of programming languages (Python v3, R v3.3, Scala v2.12) and computational libraries and frameworks (e.g. Apache Spark v2.4.5, GCTA v1.93) and stored in Parquet format when possible. The resulting 2TB of inferences are loaded into ClickHouse v20.1.4.14 and ElasticSearch v5.6.16 (Supplemental Figure S1). These two services are configured, loaded and optimised in GCP. More importantly, they are released publicly to users interested in creating their own instances of the Genetics Portal. The API is also available at https://genetics-api.opentargets.io and implemented using GraphQL v2.0.0, Play Framework v2.7.3, Slick framework v3.3.2 and Sangria v2.0.0. The web application is available at https://genetics.opentargets.org and utilises React v16.8 as well as a number of javascript libraries with emphasis on D3.js v5.5 for custom interactive visualisations. The large amount of data also introduces challenges for some user interface components. To build the locus plot (Supplemental Figure S2) for example, we use a canvas element as it is not possible to accomplish the visualisation with regular DOM nodes. To ensure global access, Open Targets Genetics is deployed on GCP across three different regions: in Europe (Belgium zone), United States (South Carolina zone) and Asia (Tokyo zone) (Supplemental Figure S1). Each regional deployment is the same and globally balanced. All code is open source and accessible in the repositories listed in Supplementary Table S1.

### Navigating the available features within the Open Targets Genetics Portal user interface

**Figure 2.**
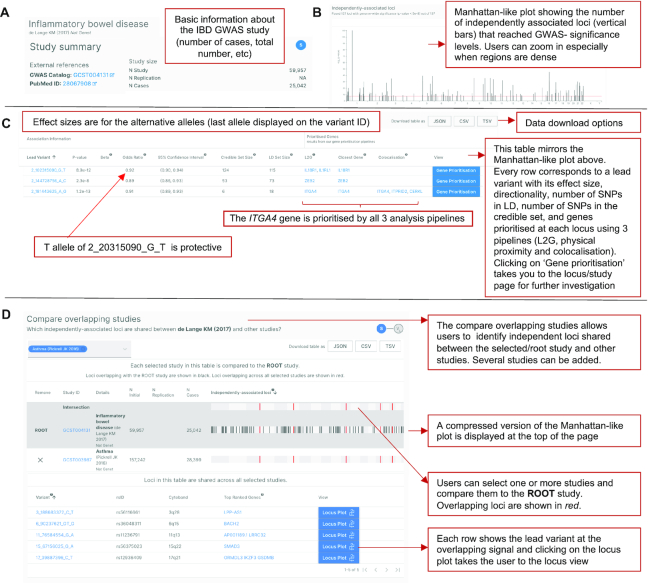
Study-trait page. The study-trait page in the Open Targets Portal for the associated trait inflammatory bowel disease from the study (22). Abbreviations: IBD; inflammatory bowel disease, ID; identifier, GWAS; Genome-wide association study, LD; linkage disequilibrium, L2G; locus-to-gene, SNP; single nucleotide polymorphism.

**Figure 3.**
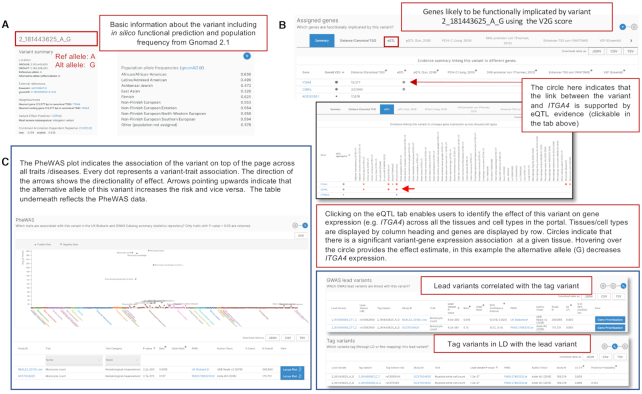
Variant page. (**A**) Each variant in the Open Targets Genetics Portal is represented with a standardised identifier of: chromosome_chromosomal location (Build GRCh38)_reference allele_alternative allele. Overview information is provided for the variant at the top of the page, such as allele frequency and predicted functional consequence. (**B**) Assigned genes using the Variant to Gene score and expression data evidence for the link between the variant and gene. (**C**) PheWAS plot and data. Abbreviations: eQTL; expression quantitative trait loci, LD; linkage disequilibrium, PheWAS; phenome wide association study, V2G; variant to gene.

**Figure 4.**
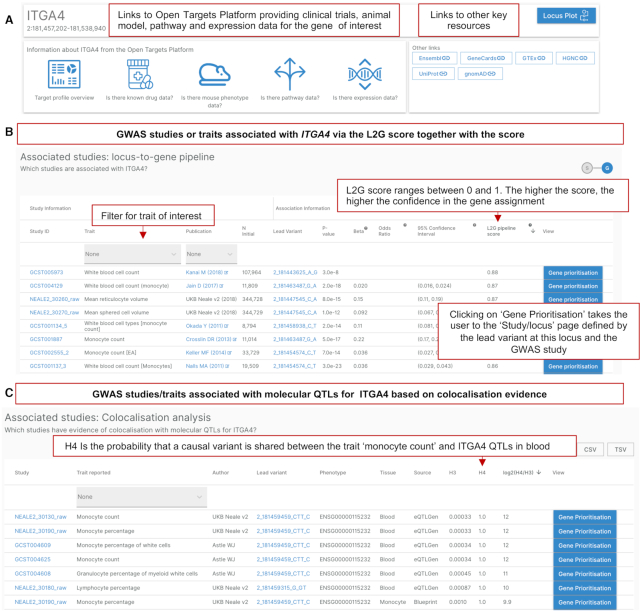
Gene page. (**A**) Overview of the gene and chromosome location (Build GRCh38), with links to the Open Targets Platform and other key data resources for relevant information. (**B**) GWAS studies or traits associated with the gene from the Locus-to-Gene analysis pipeline. (**C**) GWAS studies or traits associated with molecular quantitative trait loci for the gene, based on colocalization analysis evidence. Abbreviations: GWAS; Genome-wide association study, QTLs; quantitative trait loci, L2G; locus-to-gene.

**Figure 5.**
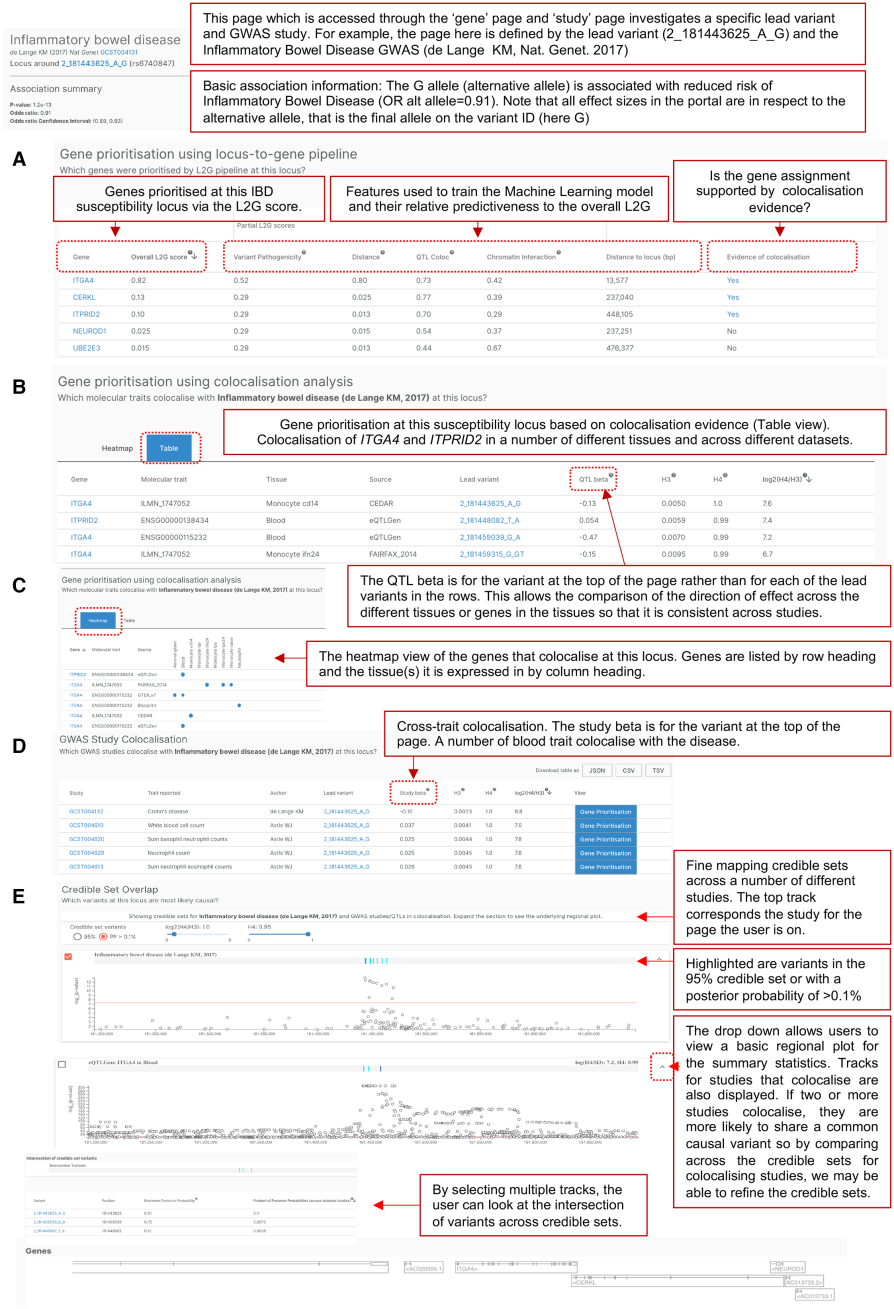
Study-locus page. An example of the gene prioritisation page for the lead variant 2_181443625_A_G and the inflammatory bowel disease GWAS^24^. Gene prioritisation results from (**A**) the locus-to-gene analysis pipeline and (**B**) colocalisation analysis. (**C**) A heatmap view of genes that colocalise at this locus. (**D**) GWAS study colocalisation. (**E**) Credible set overlap. Abbreviations: GWAS; Genome-wide association study, IBD; inflammatory bowel disease, L2G; locus-to-gene, OR; odds ratio, QTLs; quantitative trait loci.

Users can utilise the Genetics Portal to address a range of biological questions (Supplemental Figure S3). Depending on their question, users may start with a search for a gene, variant (genomic position or dbSNP reference sequence ID) or a trait/disease of interest. The data visualisations and tools provided on these pages allow users to navigate and explore the information to help answer their biological questions (Figures 2–5, Supplemental Figures S2 and S4).

Figure 2 provides an example of the information displayed on the study-trait page. The first section provides an overview of the study sample size and links to the original publication (Figure 2A). The second section provides a Manhattan-like plot that displays the independently associated variants that reached GWAS significance threshold (Figure 2B). A table provides the effect sizes of the alternative allele and the genes likely to be involved at each susceptibility locus prioritised by the statistical genetics analysis pipelines (colocalisation, L2G score, and closest gene) (Figure 2C). Using the ‘compare studies’ button on the study page, users can also compare the Manhattan plot for the root study with Manhattan plots for other studies to identify shared independent loci across the genome (https://genetics-docs.opentargets.org/how-to-use-open-targets-genetics-starting-with/multiple-traits-or-diseases) (Figure 2D).

Figure 3 provides an example of the information displayed on the variant page. If a user is interested in the genes that are likely to be functionally implicated by a given variant in a disease-agnostic way, the first section ‘Assigned genes’ displays the results from the Variant to Gene (V2G) pipeline, with a list of genes prioritised by their V2G score and the functional genomics evidence that supports this connection (Figure 3B). If gene expression data supports this connection, it is possible to view the effect of the variant on gene expression together with the directionality of effect and the tissue/cell type in which the effect is observed. If the user is interested in the traits/diseases associated with a given variant, then a PheWAS plot together with the directionality of effect of the alternative allele across different phenotypes is displayed (Figure 3C).

Figure 4 provides an example of the information displayed on the gene page. Searching for a gene of interest (in this case *ITGA4*), the user can find links to further information in the Open Targets Platform such as information about drugs in clinical trials that target this gene, animal model phenotype data, pathway data and expression data, along with links to other key resources. In order to identify traits and diseases associated with a given gene, the user can access the output from two statistical genetics analysis pipelines: the L2G analysis (Figure 4B) and the colocalisation (Figure 4C). For the L2G score, the traits prioritised can be ranked by their L2G score varying between 0 and 1, with 1 being the highest confidence trait-gene assignment at a given locus. To identify other causal gene assignments at a given susceptibility locus, the button ‘gene prioritisation’ takes the user to the study-locus page defined by a lead variant and a GWAS study.

Figure 5 provides an example of the information displayed on the study-locus page. In this example, we are investigating an Inflammatory Bowel Disease susceptibility locus defined by 2_181443625_A_G where the alternative allele (G) is protective (22). The first section (Figure 5A) displays the genes prioritised at this locus by the L2G analysis pipeline, with a breakdown of the individual predictors to show the score each gene would receive in a model including a single predictor category. The second section displays the genes prioritised at this locus using the colocalisation pipeline as a table (Figure 5B) or as a heatmap (Figure 5C). The third section displays GWAS traits and diseases that colocalise at this locus (Figure 5D). The final section provides the set of likely causal variants (credible sets) at this locus together with a regional plot view and enables the user to look at credible sets overlap between this study and other GWAS and QTL studies that colocalise at this locus (Figure 5E).

### Programmatic access to the Genetics Portal

Users can access the Genetics Portal data in different programmatic ways depending on their requirements and preference: downloading data via EMBL-EBI’s FTP download service or Google Cloud Storage service; running systematic analyses on datasets using Google Cloud BigQuery service; and executing specific queries with various GraphQL API endpoints (Figure 1E). Details on data download access is available at https://genetics-docs.opentargets.org/technical-pipeline/data-download while information on GraphQL API access is available at https://genetics-docs.opentargets.org/technical-pipeline/graphql-api.

### Users of the Genetics Portal

The public availability and wide applicability of the genetics-trait association data provided can serve a wide range of users with different expertise. As the portal software is also open source, users can install their own instance and integrate their own data. In addition, the gene prioritisation data using the L2G score from the Open Targets Genetics Portal feeds into the genetic association evidence to support target – disease associations in the Open Targets Platform (5).

## DISCUSSION

We have built an integrated system for incorporating GWAS, eQTL, pQTL and epigenetics data resources together and provide statistical genetics analysis of this data within an easy-to-navigate portal, which requires compatibility of data features (for example genes are mapped to Ensembl gene IDs and variants to chromosomal position on genome build GRCh38). To integrate genetic and functional genomics data from the wide array of disease association studies and functional genomics datasets, a major challenge is the mapping of traits from different data resources to a standardised terminology to allow systematic aggregation of associations linked to the same underlying trait or disease. We have used the Experimental Factor Ontology (EFO) to map disease terms across data resources and have added new EFO terms where required. This involves extensive manual review for each new data resource added to ensure automatic mappings are correct. Another challenge is the availability and format of data, and the requirement of making human genetic and disease data publicly available within an ethical framework. This limits the resources and types of data we are able to bring into the portal, utilising datasets that have permissions for public dissemination, have been published previously, or providing summary statistics of cohort-level information. Some of the statistical genetic analyses we carry out utilise data sets such as LD matrices that are kept for internal use only, provided to us by the data resource but that contain individual-level data that cannot be shared publicly. Availability of suitable LD matrices for non-European populations is also a limitation preventing application of all analyses across all available data. The data analysis pipelines pose challenges, such as the length of time it takes to run co-localisation (currently a month) due to the amount of data involved, and which we are improving to gain faster response times. The strength of the Open Targets Genetics Portal is that we overcome these challenges for the benefit of our users.

In addition to backend improvements which will enable more frequent release cycles, future work focuses on the integration of available data sets for more diverse populations. This includes international biobanks and population studies as they become publicly available, as well as developing LD matrices for non-European populations to allow GWAS catalog and Biobank data for these populations to be analysed. We plan to integrate COVID-19 infection susceptibility genetic study results as these become available, to aid in the drug discovery effort for the global pandemic by providing supporting genetic evidence for key human target genes. We also plan to develop existing features, such as expanding the colocalisation view to be genome-wide.

In conclusion, with the advent of GWAS, exome and whole genome sequencing and a growing number of national genomics population studies and biobanks, there is now a huge volume of human genetic and functional data linked to disease traits. The challenge is integration and systematic analysis of this data to enable robust statistical associations and prioritisation of genes underlying disease causation. The Open Targets Genetics Portal addresses these challenges and provides the results in a unique open platform which can be queried programmatically or via an informative user interface. This enables users to address a wide range of research questions, and provides underlying evidence to aid drug discovery.

## DATA AVAILABILITY

The Open Targets Genetics Portal is available at https://genetics.opentargets.org/. The entire codebase, including data integration and analyses pipelines and user interface, is open source, hosted on GitHub at https://github.com/opentargets, and licensed under the Apache License Version 2.0 (APLv2).

## Supplementary Material

gkaa840_Supplemental_File

## Notes

Present address: Eliseo Papa, R&D IT, AstraZeneca, 1 Francis Crick Avenue, Cambridge CB2 0RE, UK.

## References

[1] Hay M., Thomas D.W., Craighead J.L., Economides C., Rosenthal J. Clinical development success rates for investigational drugs. Nat. Biotechnol. 2014; 32:40–51. 24406927 10.1038/nbt.2786

[2] DiMasi J.A., Grabowski H.G., Hansen R.W. Innovation in the pharmaceutical industry: new estimates of R&D costs. J. Health Econ. 2016; 47:20–33. 26928437 10.1016/j.jhealeco.2016.01.012

[3] Nelson M.R, Tipney H., Painter J.L., Shen J., Nicoletti P., Shen Y., Floratos A., Sham P.C., Li M.J., Wang J. et al. The support of human genetic evidence for approved drug indications. Nat. Genet. 2019; 47:856–860. 10.1038/ng.3314 26121088

[4] King E.A., Davis J.W., Degner J.F. Are drug targets with genetic support twice as likely to be approved? Revised estimates of the impact of genetic support for drug mechanisms on the probability of drug approval. PLos Genet. 2019; 15:e1008489. 31830040 10.1371/journal.pgen.1008489 PMC6907751

[5] Carvalho-Silva D., Pierleoni A., Pignatelli M., Ong C., Fumis L., Karamanis N., Carmona M., Faulconbridge A., Hercules A., McAuley E. et al. Open Targets Platform: new developments and updates two years on. Nucleic Acids Res. 2019; 47:D1056–D1065. 30462303 10.1093/nar/gky1133 PMC6324073

[6] Koscielny G., An P., Carvalho-Silva D., Cham J.A., Fumis L., Gasparyan R., Hasan S., Karamanis N., Maguire M., Papa E. et al. Open Targets: a platform for therapeutic target identification and validation. Nucleic Acids Res. 2017; 45:D985–D994. 27899665 10.1093/nar/gkw1055 PMC5210543

[7] Buniello A., MacArthur J.A.L., Cerezo M., Harris L.W., Hayhurst J., Malangone C., McMahon A., Morales J., Mountjoy E., Sollis E. et al. The NHGRI-EBI GWAS Catalog of published genome-wide association studies, targeted arrays and summary statistics 2019. Nucleic Acids Res. 2019; 47:D1005–D1012. 30445434 10.1093/nar/gky1120 PMC6323933

[8] Zhou W., Zhou W., Nielsen J.B., Fritsche L.G., Dey R., Gabrielsen M.E., Wolford B.N., LeFaive J., VandeHaar P., Gagliano S.A. et al. Efficiently controlling for case-control imbalance and sample relatedness in large-scale genetic association studies. Nat. Genet. 2018; 50:1335–1341. 30104761 10.1038/s41588-018-0184-y PMC6119127

[9] Sun B.B, Maranville J.C., Peters J.E., Stacey D., Staley J.R., Blackshaw J., Burgess S., Jiang T., Paige E., Surendran P. et al. Genomic atlas of the human plasma proteome. Nature. 2018; 558:73–79. 29875488 10.1038/s41586-018-0175-2 PMC6697541

[10] Kerimov N., Hayhurst J.D., Manning J.R., Walter P., Kolberg L., Peikova K., Samoviča M., Burdett T., Jupp S., Parkinson H. et al. eQTL Catalogue: a compendium of uniformly processed human gene expression and splicing QTLs. 2020; bioRxiv doi:29 January 2020, preprint: not peer reviewed 10.1101/2020.01.29.924266. PMC8423625 34493866

[11] Võsa U., Claringbould A., Westra H.-.J., Bonder M.J., Deelen P., Zeng B., Kirsten H., Saha A., Kreuzhuber R., Kasela S. et al. Unraveling the polygenic architecture of complex traits using blood eQTL metaanalysis. 2018; bioRxiv doi:19 October 2018, preprint: not peer reviewed 10.1101/447367.

[12] Jung I., Schmitt A., Diao Y., Lee A.J., Liu T., Yang D., Tan C., Eom J., Chan M., Chee S. et al. A compendium of promoter-centered long-range chromatin interactions in the human genome. Nat. Genet. 2019; 51:1442–1449. 31501517 10.1038/s41588-019-0494-8 PMC6778519

[13] Andersson R., Gebhard C., Miguel-Escalada I., Hoof I., Bornholdt J., Boyd M., Chen Y., Zhao X., Schmidl C., Suzuki T. et al. An atlas of active enhancers across human cell types and tissues. Nature. 2014; 507:455–461. 24670763 10.1038/nature12787 PMC5215096

[14] Thurman R.E, Rynes E., Humbert R., Vierstra J., Maurano M.T., Haugen E., Sheffield N.C., Stergachis A.B., Wang H., Vernot B. et al. The accessible chromatin landscape of the human genome. Nature. 2012; 489:75–82. 22955617 10.1038/nature11232 PMC3721348

[15] Yates A.D, Achuthan P., Akanni W., Allen J., Allen J., Alvarez-Jarreta J., Amode M.R., Armean I.M., Azov A.G., Bennett R. et al. Ensembl 2020. Nucleic Acids Res. 2020; 48:D682–D688. 31691826 10.1093/nar/gkz966 PMC7145704

[16] Karczewski K.J, Francioli L.C., Tiao G., Cummings B.B., Alföldi J., Wang Q., Collins R.L., Laricchia K.M., Ganna A., Birnbaum D.P. et al. The mutational constraint spectrum quantified from variation in 141,456 humans. Nature. 2020; 581:434–443. 32461654 10.1038/s41586-020-2308-7 PMC7334197

[17] Malone J., Holloway E., Adamusiak T., Kapushesky M., Zheng J., Kolesnikov N., Zhukova A., Brazma A., Parkinson H. Modeling sample variables with an Experimental Factor Ontology. Bioinforma. Oxf. Engl. 2010; 26:1112–1118. 10.1093/bioinformatics/btq099 PMC2853691 20200009

[18] Yang J., Ferreira T., Morris A.P., Medland S.E., Madden P.A.F., Heath A.C., Martin N.G., Montgomery G.W. Genetic Investigation of ANthropometric Traits (GIANT) Consortium,DIAbetes Genetics Replication And Meta-analysis (DIAGRAM) Consortium et al. Conditional and joint multiple-SNP analysis of GWAS summary statistics identifies additional variants influencing complex traits. Nat. Genet. 2012; 44:369–375. 22426310 10.1038/ng.2213 PMC3593158

[19] Wakefield J. Bayes factors for genome-wide association studies: comparison with P-values. Genet. Epidemiol. 2009; 33:79–86. 18642345 10.1002/gepi.20359

[20] Farh K.K.-H, Marson A., Zhu J., Kleinewietfeld M., Housley W.J., Beik S., Shoresh N., Whitton H., Ryan R.J., Shishkin A.A. et al. Genetic and epigenetic fine mapping of causal autoimmune disease variants. Nature. 2015; 518:337–343. 25363779 10.1038/nature13835 PMC4336207

[21] McLaren W., Gil L., Hunt S.E., Riat H.S., Ritchie G.R., Thormann A., Flicek P., Cunningham F. The ensembl variant effect predictor. Genome Biol. 2016; 17:122. 27268795 10.1186/s13059-016-0974-4 PMC4893825

[22] de Lange K.M, Moutsianas L., Lee J.C., Lamb C.A., Luo Y., Kennedy N.A., Jostins L., Rice D.L., Gutierrez-Achury J., Ji S.G. et al. Genome-wide association study implicates immune activation of multiple integrin genes in inflammatory bowel disease. Nat. Genet. 2017; 49:256–261. 28067908 10.1038/ng.3760 PMC5289481

