# Supplementary material for: Open Targets Genetics: systematic identification of trait-associated genes using large-scale genetics and functional genomics
Source: Nucleic Acids Res. 2020 Oct 12;49(D1):D1311–20. doi: 10.1093/nar/gkaa840 (PMC7778936; doi:10.1093/nar/gkaa840)
Supplement: gkaa840_Supplemental_File [file gkaa840_supplemental_file.docx]

**SUPPLEMENTARY DATA**

**Open Targets Genetics: Systematic identification of trait-associated genes using large-scale genetics and functional genomics**

Maya Ghoussaini^1,2^, Edward Mountjoy^1,2^, Miguel Carmona^2,3^, Gareth Peat^2,3^, Ellen M. Schmidt^1,2^, Andrew Hercules^2,3^, Luca Fumis^2,3,^ Alfredo Miranda^2,3,^, Denise Carvalho-Silva^2,3,^ Annalisa Buniello^2,3^, Tony Burdett^2,3^, James Hayhurst^2,3^, Jarrod Baker^2,3^, Javier Ferrer^2,3,^, Asier Gonzalez-Uriarte^2,3^, Simon Jupp^2,3^, Mohd Anisul Karim^1,2^, Gautier Koscielny^2,7^, Sandra Machlitt-Northen^2,7^, Cinzia Malangone^2,3^, Zoe May Pendlington^2,3^, Paola Roncaglia^2,3^, Daniel Suveges^2,3^, Daniel Wright^1,2^, Olga Vrousgou^2,3^, Eliseo Papa^2,4,8^, Helen Parkinson^2,3^, Jacqueline A. L. MacArthur^3^, John A. Todd^6^, Jeffrey C. Barrett^1,2^, Jeremy Schwartzentruber^1,2^, David G. Hulcoop^2,7^, David Ochoa^2,3^, Ellen M. McDonagh^1,3^, Ian Dunham^1,2,3,*^

1. Wellcome Sanger Institute, Wellcome Genome Campus, Hinxton, Cambridgeshire CB10 1SA, UK
2. Open Targets, Wellcome Genome Campus, Hinxton, Cambridgeshire CB10 1SD, UK
3. European Molecular Biology Laboratory, European Bioinformatics Institute (EMBL-EBI), Wellcome Genome Campus, Hinxton, Cambridgeshire CB10 1SD, UK
4. Systems Biology, Biogen, Cambridge, MA, 02142, USA.
5. Integrative Biology, Internal Medicine Research Unit, Pfizer Worldwide Research, Development and Medical, Cambridge, MA 02139, USA
6. Wellcome Centre for Human Genetics, Nuffield Department of Medicine, NIHR Oxford Biomedical Research Centre, University of Oxford, Roosevelt Drive, Oxford, OX3 7BN
7. GlaxoSmithKline plc, GSK Medicines Research Centre, Gunnels Wood Road, Stevenage, SG1 2NY, UK
8. Current address: R&D IT, AstraZeneca, 1 Francis Crick Avenue, Cambridge CB2 0RE, UK

**Contents**

[**Supplementary Figure 1: Open Targets Genetics Infrastructure** 3](#_Toc48321813)

[**Supplementary Figure 2: Locus Plot** 4](#_Toc48321814)

[**Supplementary Figure 3: User journey and biological questions** 6](#_Toc48321815)

[**Supplementary Figure 4: Additional annotation for the Gene Page** 7](#_Toc48321816)

[**Supplementary Table 1: List of Open Targets Genetics Portal repositories** 9](#_Toc48321817)

# **Supplementary Figure 1: Open Targets Genetics Infrastructure**

**
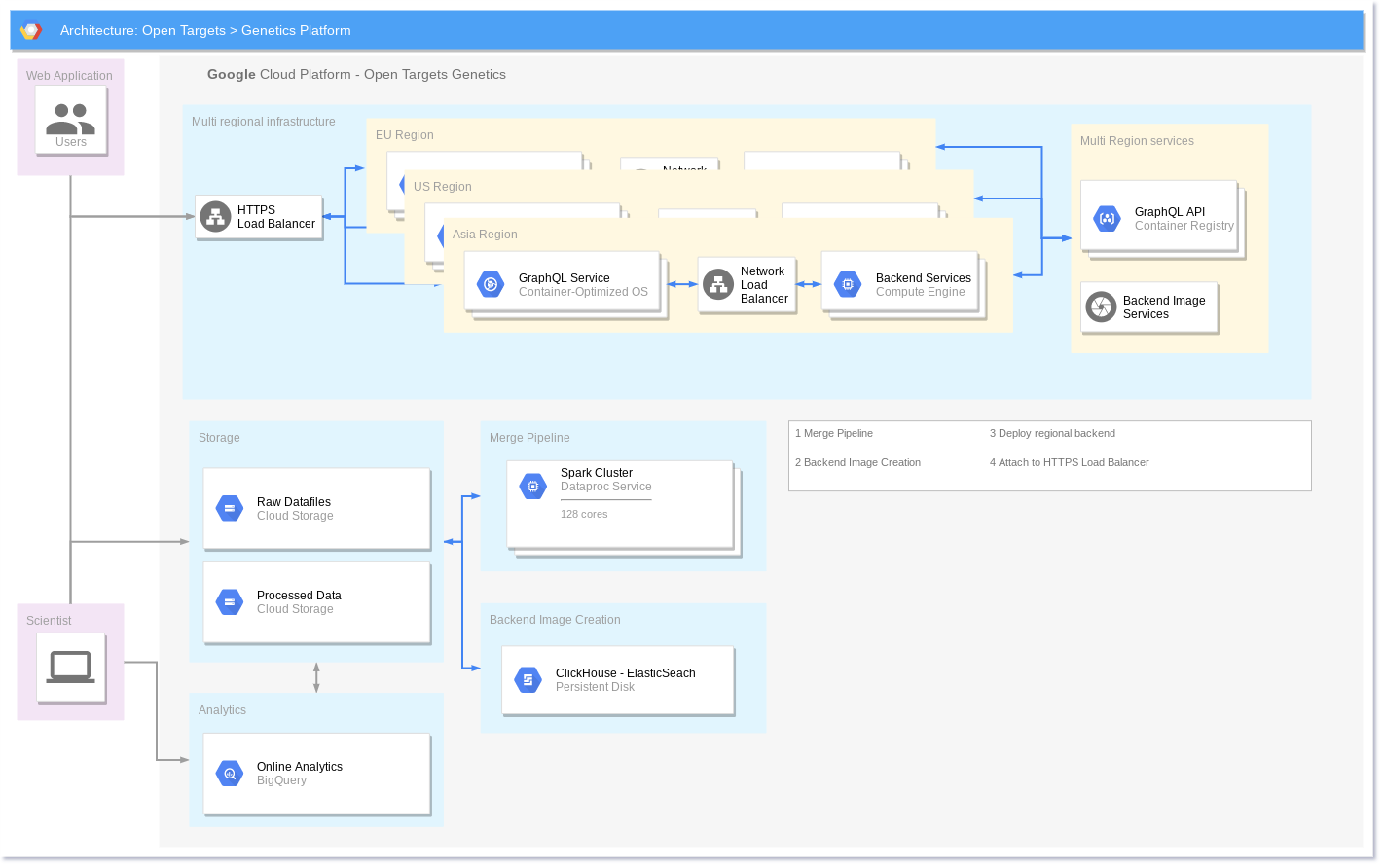
**

# **Supplementary Figure 2: Locus Plot**

**
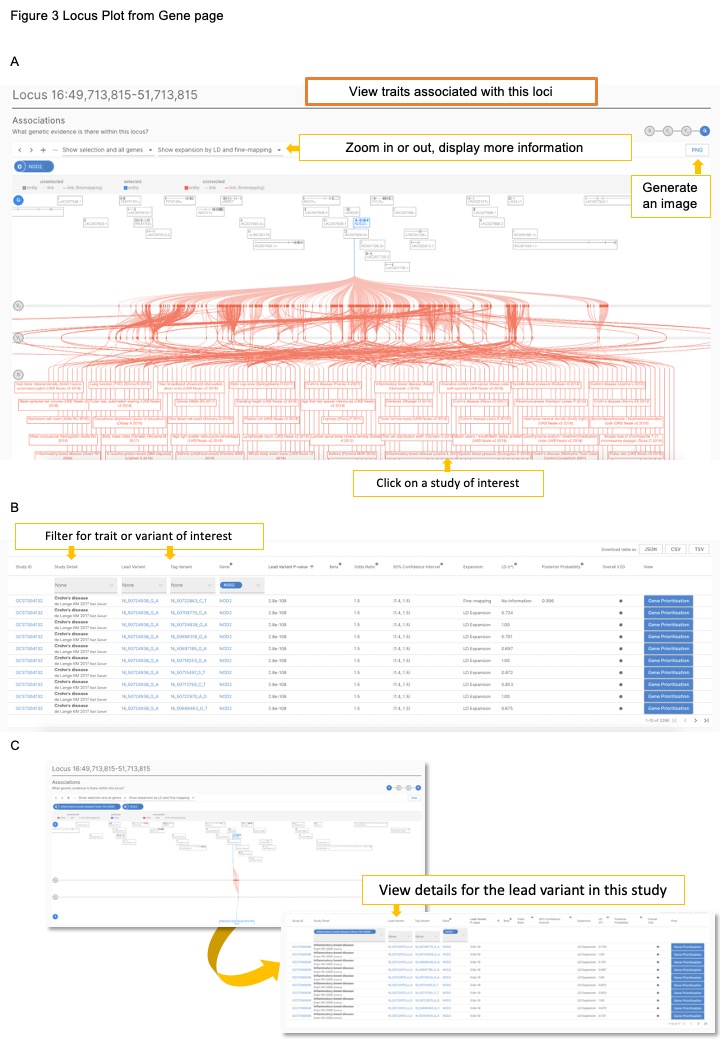
**

**Supplementary Figure 2 Legend: Locus Plot**

(A) The ‘Locus Plot’ button found on gene and variant pages leads the user to view the gene region in the context of the genome, with a map of the variant-trait associations that have been linked to this region. (B) Details of the associations are displayed in a filterable and downloadable table below the map. (C) By clicking on a study of interest on the map, the variants identified in this study and associated with the locus are narrowed down. The locus plot tool allows the user to extensively explore the region beyond the gene of interest, by deselecting the gene of interest, selecting to view all associated locus, and zooming in or out of the genome.

# **Supplementary Figure 3: User journey and biological questions**


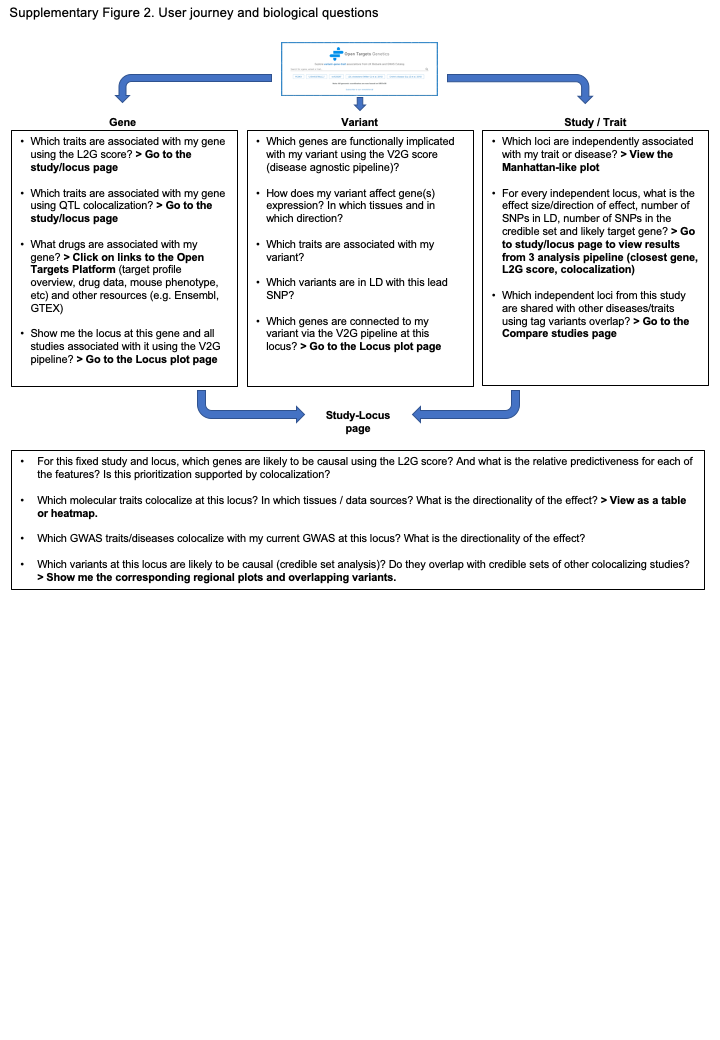


# **Supplementary Figure 4: Additional annotation for the Gene Page**


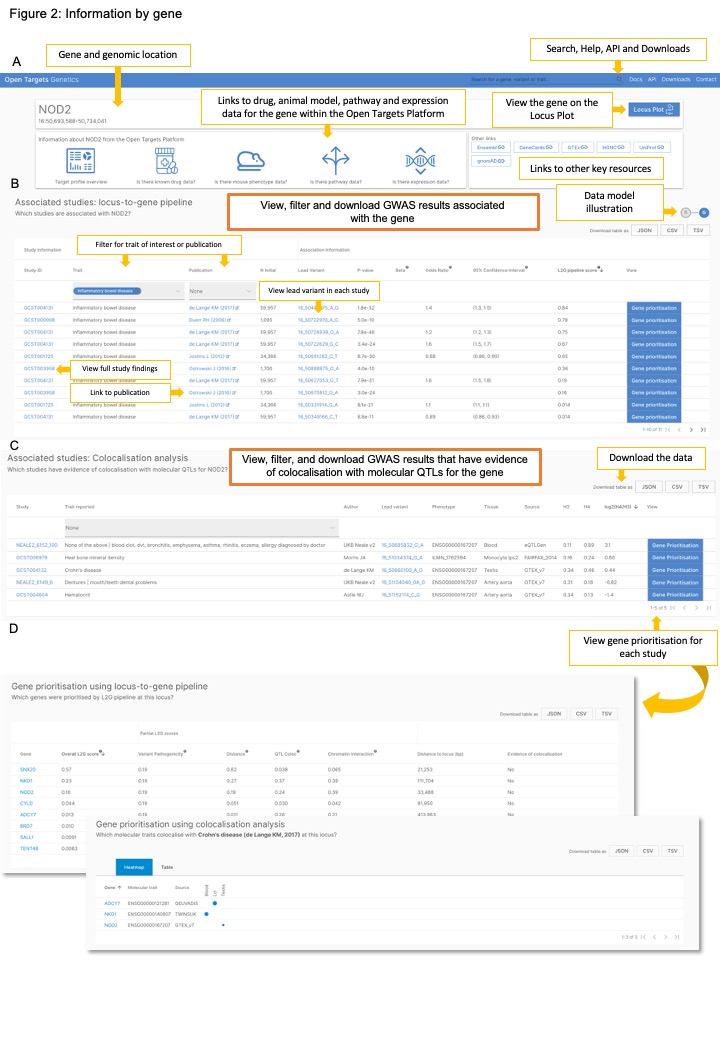


**Supplementary Figure 4 Legend: Information by gene**

Searching for a gene, the user will reach the gene profile page (as exemplified for the *NOD2* gene). (A) At the top of the gene profile page, the user can find link outs to further information in the Open Targets Platform such as drug information linked to the gene as a target, animal model phenotype data, pathway data and expression data, along with links to other key databases. (B) The user can view, filter and download results from GWAS that have been associated with the gene of interest by the locus-to-gene analysis pipeline. The results can be sorted by the evidence level based upon the locus-to-gene score, with 1 being the highest confidence that the locus identified in the study is associated with the gene. Key information for each study is provided, such as the number of samples, statistics for the trait and lead variant association, and a link to the original publication. The locus-to-gene association studies table can be filtered for a trait of interest or a particular study. (C) The second table displayed on the gene page displays associated GWAS based on colocalisation with expression quantitative trait loci (eQTLs) to provide evidence for an association with the gene. Details for the study and eQTL data are displayed. In both tables, links are provided to the full study findings and to lead variant pages within the Genetics Portal, and the data is available as a download in three different formats (JSON, CSV, TSV). (D) For both the locus-to-gene and colocalisation analyses, a link to view the prioritisation of associated genes the selected locus result is provided.

# **Supplementary Table 1: List of Open Targets Genetics Portal repositories**

| **a) Data ingest and analysis** |  |
| --- | --- |
| **Repository *https://github.com/opentargets/...*** | **Description** |
| genetics-sumstat-data | Workflow to process summary statistics |
| genetics-sumstat-harmoniser | GWAS summary statistics harmonisation pipeline |
| genetics-variant-annotation | Variant index generation |
| genetics-v2g-data | Cis-regulatory data pipeline for variant-to-gene assignment |
| genetics-v2d-data | Variant-to-disease association tables pipeline |
| genetics-finemapping | Fine-mapping analysis |
| genetics-colocalisation | Colocalsation data analysis |
| genetics-gold-standards | Gold standards of high confidence gene-loci associations |
| genetics-l2g-scoring | Locus2Gene machine learning scoring pipeline |
|  |  |
| **b) Infrastructure, API and web application** |  |
| **Repository *https://github.com/opentargets/...*** | **Description** |
| genetics-pipe | Extract-Transform-Load (ETL) pipeline |
| genetics-backend | Back-end infrastructure |
| genetics-api | GraphQL API (available at [genetics-api.opentargets.io](http://genetics-api.opentargets.io/)) |
| genetics-app | React web application (available at [genetics.opentargets.org](http://genetics.opentargets.org/)) |
